# Contribution of *Streptococcus pseudopneumoniae* and *Streptococcus salivarius* to vocal fold mucosal integrity and function

**DOI:** 10.1242/dmm.050670

**Published:** 2024-07-16

**Authors:** Vlasta Lungova, Madhu Gowda, Jessica M. Fernandez, Stephanie Bartley, Anumitha Venkatraman, Federico E. Rey, Susan L. Thibeault

**Affiliations:** ^1^ Department of Surgery, Division of Otolaryngology, University of Wisconsin-Madison, Madison, WI 53792, USA; ^2^ Department of Bacteriology, University of Wisconsin-Madison, Madison, WI 53706, USA

**Keywords:** Pathogenic bacteria, Commensals, Vocal fold mucosa, Remodeling, Epithelial integrity

## Abstract

Structural changes to the vocal fold (VF) epithelium, namely, loosened intercellular junctions, have been reported in VF benign lesions. The potential mechanisms responsible for the disruption of cell junctions do not address the contribution of resident microbial communities to this pathological phenomenon. In this study, we focused on determining the relationship between *Streptococcus pseudopneumoniae* (SP), a dominant bacterial species associated with benign lesions, and *Streptococcus salivarius* (SS), a commensal bacterium, with human VF epithelial cells in our three-dimensional model of the human VF mucosa. This experimental system enabled direct deposition of bacteria onto constructs at the air/liquid interface, allowing for the assessment of bacterium–host interactions at the cellular, molecular and ultrastructural levels. Our findings demonstrate that SP disrupts VF epithelial integrity and initiates inflammation via the exported products HtrA1 and pneumolysin. In contrast, SS attaches to the VF epithelium, reduces inflammation and induces Mmp2-mediated apical desquamation of infected cells to mitigate the impact of pathogens. In conclusion, this study highlights the complexity of microbial involvement in VF pathology and potential VF mucosal restoration in the presence of laryngeal commensals.

## Teaser

**Summary:** *Streptococcus pseudopneumoniae* disrupts vocal fold epithelial integrity and function via the exported products HtrA1 and pneumolysin. These effects are mitigated in the presence of *Streptococcus salivarius*, a laryngeal commensal.

## INTRODUCTION

The human larynx and vocal folds (VFs) are situated at the crossroads between the gastrointestinal and respiratory systems. It is the narrowest point of the airway and a point of great airway turbulence where deposition of inhaled and sometimes ingested challenges is high. The VF mucosa forms the intersecting barrier between the host organism and the external environment. It is covered by stratified squamous epithelium that serves as the first level of innate immunological defenses against all potential environmental assaults or antigens (Levendoski et al., 2014). This protective barrier is maintained through various mechanisms. First, the VF epithelium is compact, as it is ‘sealed’ together by cell junctions preventing small molecules and/or microorganisms from passing into the lamina propria (Leydon et al., 2014). Second, epithelial cells secrete mucus that entraps microbes and facilitates their extrusion through mucociliary clearance. Lastly, VF epithelial/mucosal cells are capable of activating immune cells via cytokine production to fight infection. When these protective mechanisms are disrupted, the mucosa becomes vulnerable to further damage and pathogen invasion (Leydon et al., 2014).

Benign VF lesions are common causes of hoarseness and voice disorders that have been related to disrupted mucosal homeostasis. Structural changes to the VF epithelium, namely, loosened intercellular junctions, and dilated intercellular spaces have been reported in VF nodules, polyps (Kotby et al., 1988; Mostafa et al., 2017), Reinke's edema (Pastuszek et al., 2003) and granulomas (Martins et al., 2009). The potential mechanisms for the disruption of epithelial cell junctions are limited and do not address the contribution of resident microbial communities to this pathological phenomenon. Recent evidence suggests that a possible etiology and/or progression of benign VF diseases can be associated with *Streptococcus pseudopneumoniae* (SP), which has been found to be a dominant bacterial species present in lesion samples, at the expense of commensals, specifically, *Streptococcus salivarius* (SS) (Jetté et al., 2016; Hanshew et al., 2014). SP has been recognized as an opportunistic pathogen due to its links to pulmonary exacerbations and chronic respiratory diseases (Dupont et al., 2020). Phylogenetic analysis of SP and its closest relatives from the mitis group has revealed that SP is an intermediate species between *Streptococcus pneumoniae*, a pathogen causing acute pneumococcal infections, and *Streptococcus mitis*, which is prevalent in the normal microbiota of the oropharynx, with low virulence and pathogenicity (Dupont et al., 2020). Whole-genome sequencing has further demonstrated that SP possesses several pneumococcal virulence genes in its core genome, which are also present in *S. pneumoniae*, but absent in *S. mitis* (Garriss et al., 2019; Wen et al., 2014). These genes encode serine proteases, such as a high-temperature requirement protease A1 (HtrA1), that cleave host cell–cell adhesion molecules, such as E-cadherin (E-cad, encoded by *CDH1*), and bacterial toxins, such as pneumolysin (Ply), which perforates cell membranes and promotes mucosal inflammation (Rai et al., 2016). In contrast, there are features that set SP apart from *S. pneumoniae*. SP is a non-capsulated bacterium that lacks pneumococcal surface proteins C (PspC) and A (PspA) (Shahinas et al., 2013). Both surface proteins are anchored to the bacterial capsule and represent major adhesins that *S. pneumoniae* exploits to attach to host cell membranes to cause acute infections. Instead, SP uses its own variants of adhesion proteins, such as PspK, that are anchored to the bacterial cell wall (Garriss et al., 2019). Little is known about whether these adhesins can effectively bind to VF epithelial cells and whether SP can activate its virulent genes to induce pathological VF epithelial remodeling and inflammation.

SS is a commensal bacterium that colonizes epithelial surfaces and establishes a symbiotic relationship with the host. It is a member of the respiratory tract microbiota (Manning et al., 2016). Commensals act on the immune system of the host to induce protective responses that prevent pathogen colonization and invasion. Commensals can directly inhibit the growth of pathogens by competing for nutrients and binding sites, by secretion of anti-microbial molecules and by modulation of host immune reactions (Manning et al., 2016). In addition, commensal bacteria have the capacity to stimulate non-specific host defense mechanisms, such as shedding of the uppermost epithelial layers (Nestle et al., 2009). The impact of commensal bacteria on VF epithelial maintenance is not understood.

The aim of this study was to investigate interactions between SP and SS with human VF epithelial cells using our recently developed three-dimensional (3D) organotypic model of human VF mucosa (Lungova et al., 2019). This model recapitulates fundamental features of VF mucosal complexity observed *in vivo* and provides a realistic setting for studying bacterial pathogenicity and virulence under controlled conditions. Our findings demonstrate that the microbiota can profoundly affect key aspects of VF mucosal physiology, namely, epithelial barrier integrity, and mucosal innate immune responses, which is in line with previous investigations for other cell types, such as the skin epidermis and intestinal cells (Gong et al., 2013). SP likely causes local dilation of intercellular spaces and cell junctions and triggers inflammation primarily via the exported products HtrA1 and Ply, rather than by directly adhering to the epithelial surfaces. SS, in contrast, firmly attaches to the VF epithelium, potentially displacing SP from the epithelial surface, reduces inflammation and induces Mmp2-mediated apical desquamation of infected and damaged cells to mitigate the impact of pathogens. In conclusion, this study highlights the complexity of microbial involvement in VF pathology and potential VF mucosal restoration in the presence of laryngeal commensals.

## RESULTS

### Bacterial dose determination

To determine what bacterial dose is capable of inducing mucosal remodeling without a significant increase in host cellular toxicity, we inoculated human-engineered VF mucosae with SP and SS cultures and SS/SP co-cultures for 24 h at two doses: a low dose of 10 multiplicity of infection (MOI) and a high dose of 50 MOI. Bacterial suspensions were directly applied on the top of the constructs to maintain the air/liquid interface (A/Li). VF mucosae inoculated with plain bacterial growth medium were used as controls (Fig. S1A). We rationalized that bacterial communities associated with benign VF lesions may play a role in pathological epithelial remodeling and disease progression, yet they are unlikely to cause severe epithelial damage, which is in line with clinical observations (Mostafa et al., 2017; Kotby et al., 1988). For inoculation, we used bacterial species during the mid-logarithmic growth phase, characterized by optical density (OD) values ranging from 0.4 to 0.6 nm, which corresponded to a bacterial population of approximately (2.00-3.00)×10^8^ per 1 ml (Fig. S1B,C). After 24 h, culture media from basolateral chambers of control and experimental groups were collected and analyzed using a lactate dehydrogenase (LDH) Glo cytotoxicity assay. A standard LDH release curve shows that both bacterial doses were in the linear phase of the LDH release curve and therefore suitable for this cell toxicity assessment, as recommended by the supplier (Fig. S1D). By calculating the percentage of toxicity, we observed that experimental groups exposed to low doses displayed elevated levels of LDH, which were not statistically different from those of controls (Fig. S1E). Using high bacterial doses, we found a statistically significant increase in the percentage of toxicity [one-way ANOVA with Tukey's honestly significant difference (HSD) test; *P*=0.02498] in SP and SS alone and co-cultures in contrast to that for control VF mucosae (Fig. S1E). We primarily used low bacterial doses in our subsequent experiments as they were more physiologically relevant. However, we included results from higher doses for illustrative purposes.

### Bacterial species can interact with the host epithelium, influencing its structural integrity

**Fig. 1.**
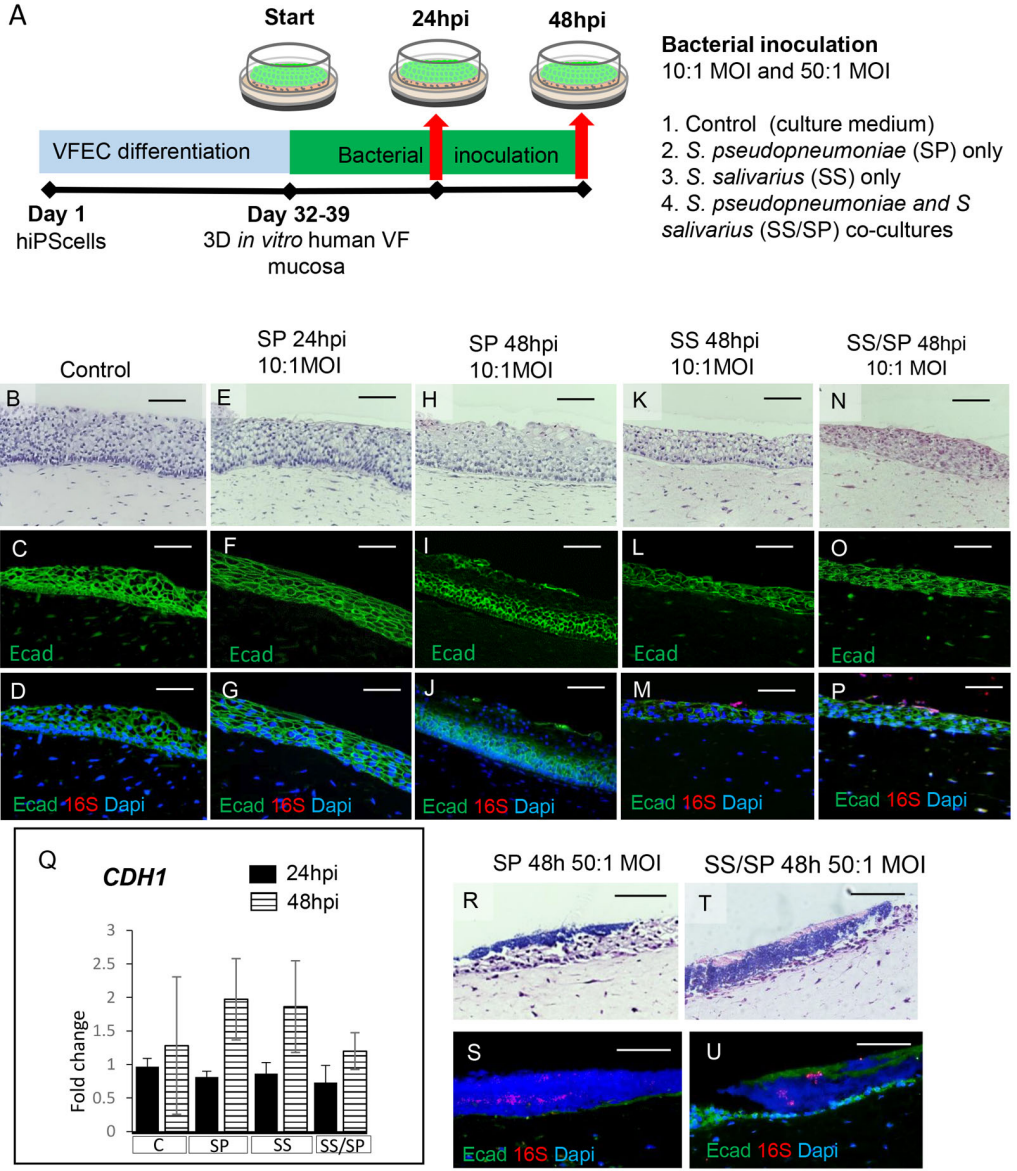
**Schematic illustration of the experimental design, and morphology and structure of vocal fold mucosa exposed to bacterial species.** (A) Schematic illustration of the experimental design. Human induced pluripotent stem cells (hiPSCs) were first differentiated into vocal fold (VF) basal progenitors that were re-seeded on collagen–fibroblast constructs to create a three-dimensional (3D) model of human VF mucosa. VF mucosae were inoculated with bacterial species between 32 and 39 days of differentiation for 24 and 48 h. We included control VF mucosae inoculated with plain bacterial growth medium (1) and three experimental groups exposed to *Streptococcus pseudopneumoniae* (SP) only (2), *Streptococcus salivarius* (SS) only (3) and co-cultures of SP and SS (4). We used two bacterial doses: 10:1 multiplicity of infection (MOI) and 50:1 MOI. (B,E,H,K,N,R,T) Hematoxylin and Eosin staining of VF mucosae in control (B) and experimental groups exposed to low bacterial doses (E,H,K,N) and high bacterial doses (R,T) for the assessment of VF epithelial morphology. (C,D,F,G,I,J,L,M,O,P,S,U) Anti-E-cad staining (green) alone and in combination with the 16S probe (red) was performed to illustrate the compactness of the VF epithelium and bacterial colonization in control (C,D) and experimental groups exposed to low bacterial doses (F,G,I,J,L,M,O,P) and high bacterial doses (S,U). Images are representative of three experiments. Scale bars: 100 µm. (Q) Expression levels of the *CDH1* gene that encodes E-cad in the control group (‘C’) and using a low bacterial dose of 10:1 MOI at both time points. Error bars represent ±s.e.m. obtained from three biological and two technical replicates. One-way ANOVA for independent or correlated samples along with Tukey's HSD test was used for statistical analysis. hpi, hours post inoculation; VFEC, vocal fold epithelial cells.

Next, we investigated how bacterial communities interact with the host VF epithelium. We inoculated engineered VF mucosae with SP and SS cultures and SS/SP co-cultures for 24 and 48 h at MOIs of 10 and 50 as mentioned above, with controls inoculated with plain bacterial growth medium (Fig. 1A). On gross examination, there were no visible indications of pathological remodeling or epithelial damage in the VF mucosae of control and low-dose-exposed groups for both time points (Fig. 1B,E,H,K,N). Nevertheless, when we stained for the cell adherence marker E-cad, we found that E-cad was absent in the apical epithelial cell layers exposed to SP, particularly at 48 h post inoculation (hpi), compared to E-cad in controls or mucosae inoculated with SP at the early 24 hpi time point (Fig. 1C,F,I). The loss of E-cad was not observed in VF mucosae inoculated with SS and SS/SP at 48 hpi (Fig. 1L,O). Next, we performed fluorescence *in situ* hybridization (FISH) using a 16S RNA probe in combination with E-cad staining to determine whether E-cad disintegration correlated with bacterial colonization. 16S FISH hybridization offers a versatile approach that, although not exclusively limited to bacterial species, has the capability to visualize bacterial adherence and penetration into the host epithelium. Notably, we did not detect a red 16S signal in controls and SP groups at 24 and 48 hpi (Fig. 1D,G,J), indicating that SP did not attach to the VF epithelial surface and/or was present in low quantities. However, we detected a red 16S signal in apical epithelial cells in SS and SS/SP co-cultures at 48 hpi (Fig. 1M,P). Moreover, in SS/SP co-cultures, 16S staining was stronger than in cultures exposed to SS only, suggesting that bacteria robustly attached to the epithelial surface when cultured together. Simultaneously, we conducted quantitative polymerase chain reaction (qPCR) targeting the *CDH1* gene that encodes the E-cad protein (Fig. 1Q). No significant disparities in the expression of *CDH1* were observed between control and experimental groups at both time points. These results indicate that the loss of E-cad in the uppermost cell layers did not occur due to a direct downregulation of *CDH1*. Instead, it likely resulted from post-translational E-cad protein modification or cleavage. With high bacterial doses (50 MOI), we observed massive VF epithelial cell damage with E-cad degradation accompanied by bacterial invasion of the epithelium (Fig. 1R-U).

**Fig. 2.**
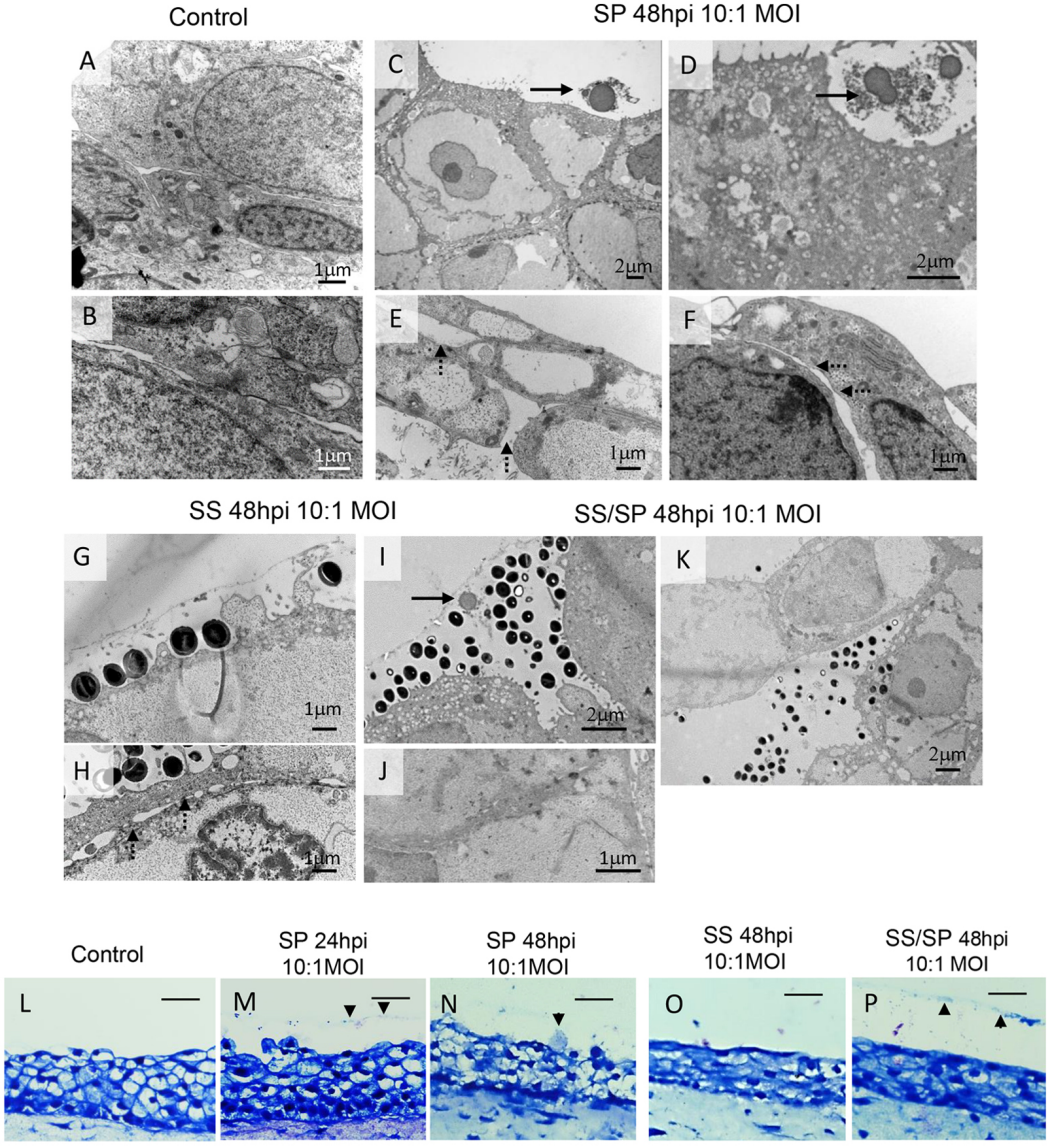
**Ultrastructure of vocal fold mucosae exposed to bacterial species and detection of biofilm.** (A,B) Ultrastructure of VF epithelial cells of control VF mucosae. (C-F) Ultrastructure of VF epithelial cells exposed to a low dose of SP at 48 h post inoculation (hpi). Black solid arrows (C,D) denote bacterial cells with their self-produced matrix. Black dashed arrows (E,F) denote loosened cell junctions and dilated intercellular spaces. (G,H) Ultrastructure of VF epithelial cells exposed to low dose of SS at 48 hpi. Bacteria can be seen firmly attached to epithelial cells. Black dashed arrows (H) denote existing cell junctions and the compact VF epithelium. (I-K) Ultrastructure of VF epithelial cells exposed to a low dose of SS/SP co-cultures 48 hpi. The black solid arrow (I) points to displacement of SP with its self-producing matrix from the epithelial surface, likely due to the robust attachment of SS. Some apical epithelial cells can undergo desquamation (K). (L-P) Giemsa staining in control samples (L), SP-alone samples at 24 hpi (M), SP-alone samples at 48 hpi (N), SS-alone samples at 48 hpi (O) and SS/SP co-cultures at 48 hpi (P). Black arrowheads in M,N,P denote positive staining of bacterial clusters. Scale bars: 50 µm. Images are representative of two independent experiments (TEM) and three independent experiments (Giemsa staining).

To provide additional confirmation of the distinct abundance and adhesion properties of SP and SS to VF epithelial cells and their potential impact on cell junctions, we performed transmission electron microscopy (TEM). In control VF mucosae, epithelial cells were tightly packed together, as expected for epithelial sheets (Fig. 2A,B). In VF mucosae inoculated with SP, few bacteria were observed in close proximity to the host epithelial surface. They did not appear to firmly adhere to the cytoplasmic membranes, but instead generated a matrix that kept bacteria close to each other (Fig. 2C,D). Intercellular spaces between adjacent epithelial cells were markedly enlarged and cell junctions loosened (Fig. 2E,F). Regarding SS, these bacteria adhered to the host cell membrane, without any discernible impact on cell junctions or dilation of intercellular spaces (Fig. 2G,H). Notably, in SS/SP co-cultures, we observed a robust adherence of SS to apical host cells, which appeared to displace SP with its self-producing matrix from the epithelial surface (Fig. 2I). Moreover, robust SS colonization likely induced shedding of apical cells, thereby maintaining the compactness of the epithelium (Fig. 2J,K). To visualize extracellular biofilm matrix on histological slides, we performed Giemsa staining (Jensen et al., 2018). We detected bacterial stains in SP cultures at 24 and 48 hpi (Fig. 2M,N) and in SS/SP co-cultures at 48 hpi (Fig. 2P); however, fewer stains were observed in SS alone (Fig. 2O) and in the controls (Fig. 2L). Collectively, these findings show that both bacterial species exhibit strategies to interact with the VF epithelium leading to pathogenic or protective outcomes.

### Assessment of bacterial cell viability during the culture conditions

**Fig. 3.**
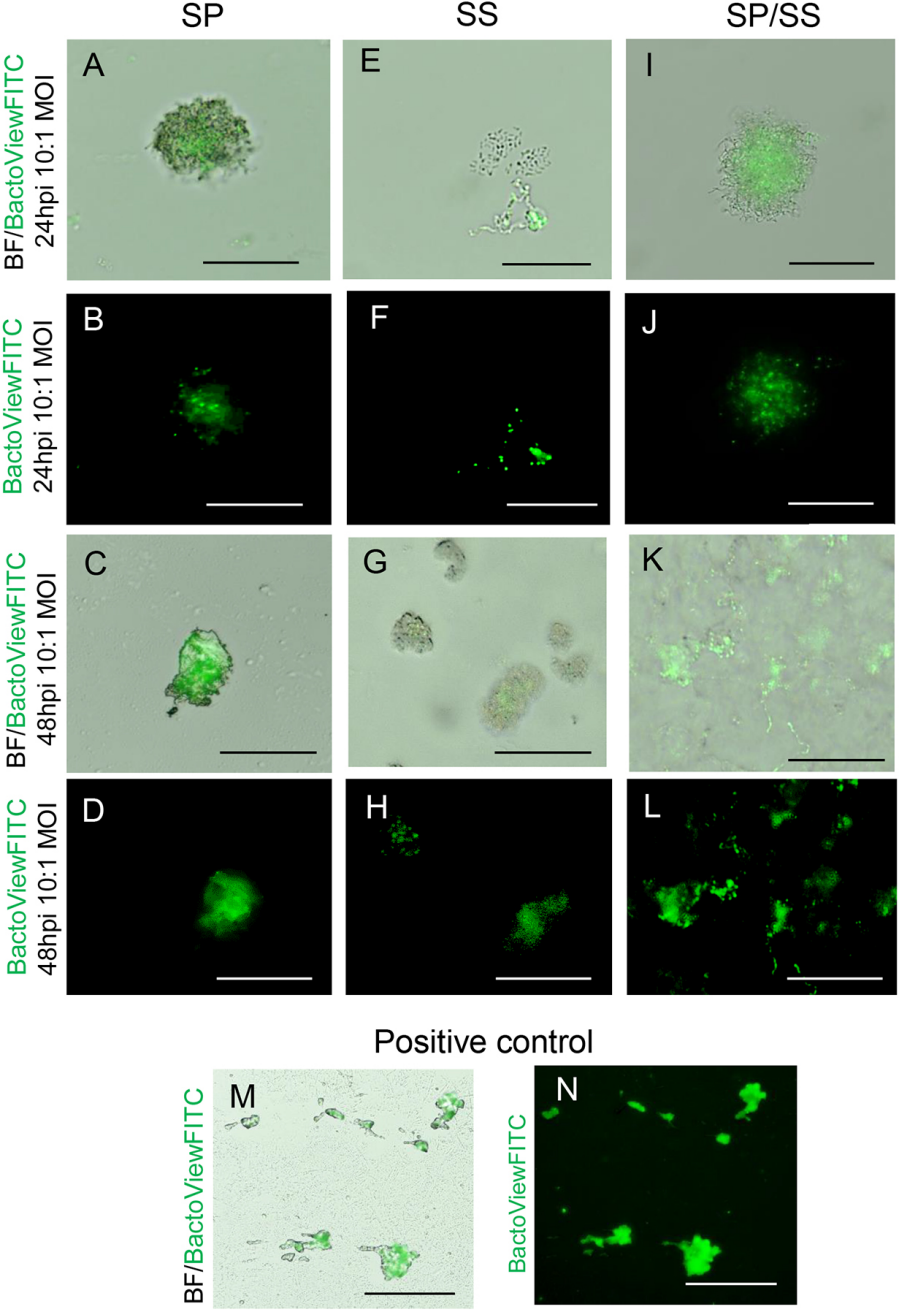
**Bacterial viability assay.** (A-D) Brightfield (BF) and BactoView FITC staining in SP cultures at 24 and 48 hpi at a dose of 10:1 MOI. (E-H) Brightfield and BactoView FITC staining in SS cultures at 24 and 48 hpi at a dose of 10:1 MOI. (I-L) Brightfield and BactoView FITC staining in SS/SP co-cultures at 24 and 48 hpi at a dose of 10:1 MOI. (M,N) Brightfield and BactoView FITC staining in heat-inactivated bacteria at a dose of 10:1 MOI. Green signals denote dead bacterial cells. Scale bars: 50 µm. Images are representative of two independent experiments.

Next, we conducted a bacterial cell viability assay for SP and SS during culturing. Given the significant differences between the A/Li and flavonoid adenine dinucleotide (FAD) medium culture conditions post inoculation versus the original culture conditions for both bacterial species, we sought to determine whether the bacteria remained viable or if the observed changes in the VF epithelium/mucosa were caused by bacterial cell lysates. During cell lysis, bacterial extracellular content become incorporated into the biofilm, which can act as a passive virulent factor (Schulze et al., 2021). To assess bacterial viability, bacterial suspensions (SP, SS and SS/SP co-cultures) at a dose of 10:1 MOI were mixed with Matrigel to immobilize the bacteria and plated on culture dishes in drops. These were allowed to solidify in an incubator at 37°C and 5% CO_2_ at least for 15 min. Subsequently, the drops were partially flooded with FAD medium to mimic A/Li conditions. After 24 and 48 hpi, we stained cell cultures with a fluorogenic DNA-binding dye (BactoView Dead Stain) for live/dead cell discrimination in bacteria. Our results showed that SP had a tendency to agglutinate during culturing, with some cells undergoing cell death at 24 h that was particularly evident at 48 hpi (Fig. 3A-D). SS bacteria, in contrast, formed chains at 24 hpi (Fig. 3E) and began to aggregate at 48 hpi (Fig. 3G). We also noted an increase in cell death with prolonged cell cultivation (Fig. 3E-H). In SS/SP co-cultures, bacterial cell cultures likely contained both species, as indicated by the visible elongated bacterial chains extending from the clustered core (Fig. 3I). We also detected an increase in cell death with prolonged cell cultivation (Fig. 3I-L). Heat-inactivated bacteria were used as a positive control for this experiment (Fig. 3M,N). These findings suggest that the physical presence of bacteria and their cellular contents can both contribute to the structural and functional changes in the VF epithelium or mucosa.

### Effects of SP and SS on the function of the protective epithelial barrier and VF mucosal defensive responses

**Fig. 4.**
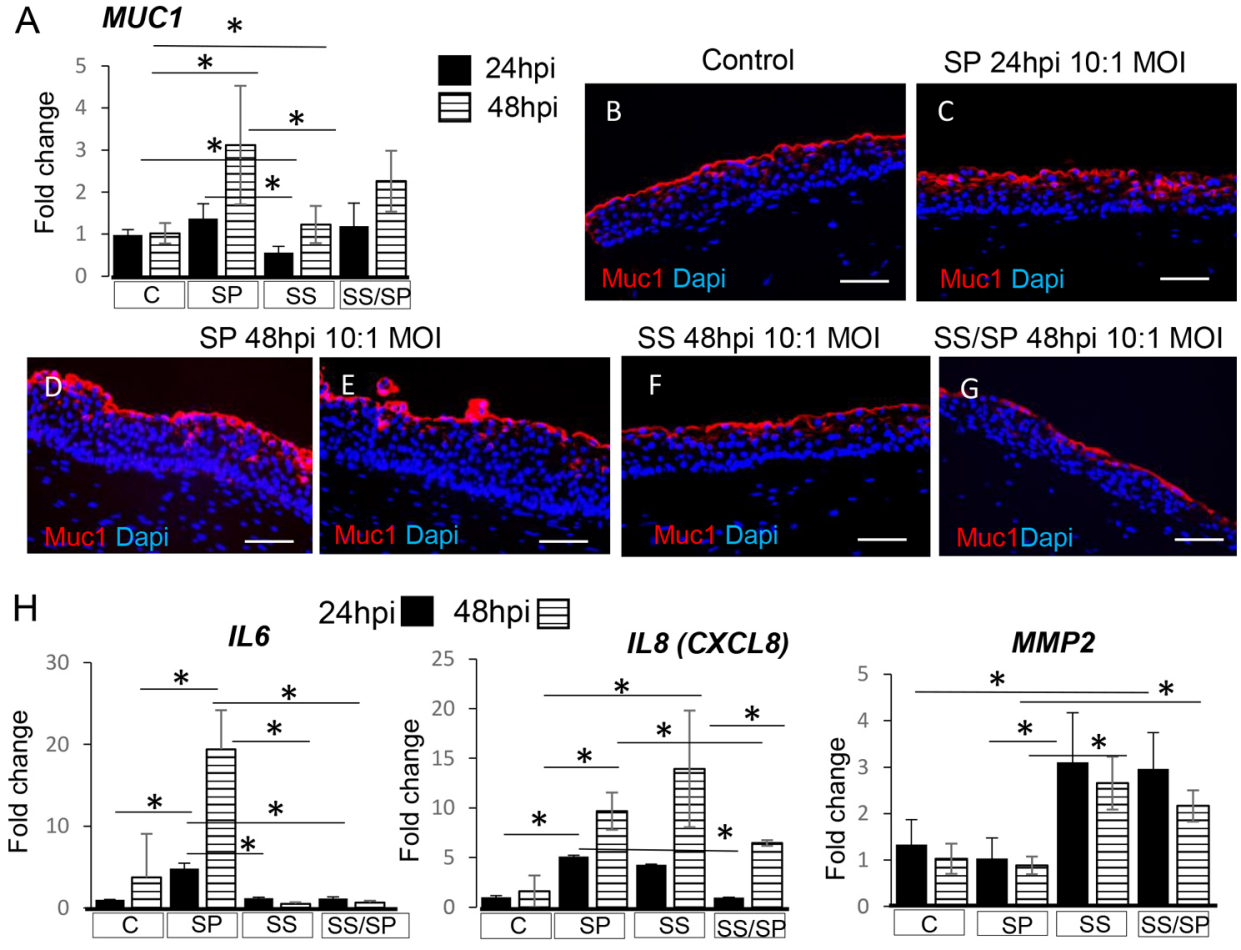
**Human vocal fold mucosal responses to bacterial species used in the study.** (A) Assessment of *MUC1* gene expression at 24 and 48 hpi. We observed significant upregulation of *MUC1* in the SP group compared to its expression in the control and SS groups (one-way ANOVA with Tukey's HSD test, *P*=0.00897 at 24 hpi and 0.004007 at 48 hpi). (B-G) Immunofluorescent anti-Muc1 staining in VF mucosal cells. Scale bars: 100 µm. Images are representative of three experiments. (H) Expression levels of *IL6* at 24 and 48 hpi. Exposure to SP significantly stimulated *IL6* expression in VF mucosal cells 24 hpi (one-way ANOVA, *P*=1.715×10^−7^) and 48 hpi (one-way ANOVA, *P*=0.00001486) compared to that in controls (Tukey's HSD, *P*=3.248×10^−7^ at 24 hpi and *P*=0.0001074 at 48 hpi), SS (Tukey's HSD, *P*=5.218×10^−7^ at 24 hpi and 0.00003834 at 48 hpi) and SS/SP (Tukey's HSD, *P*=4.9117×10^−7^ at 24 hpi and 0.00004152 at 48 hpi) co-cultures. (I) Expression levels of *IL8* at 24 and 48 hpi. For the 24 hpi time point, we detected a significant increase in *IL8* expression in SP (one-way ANOVA, *P*=2.68×10^−12^) compared to that in controls (Tukey’s HSD, *P*=3.705×10^−13^), SS groups (Tukey's HSD, *P*=0.000003323) and SS/SP co-cultures (Tukey's HSD, *P*=3.076×10^−13^). For the 48 hpi time point, we found a significant upregulation of *IL8* (one-way ANOVA, *P*=0.000139) in the SP group compared to its expression in controls (Tukey's HSD, *P*=0.00476) and in SS groups (Tukey's HSD, *P*=0.00009586), but not for SS/SP co-cultures. (J) Expression levels of *MMP2* at 24 and 48 hpi (J). We found significant upregulation of *MMP2* in the SS and SS/SP groups at 24 hpi compared to its expression in controls (one-way ANOVA, *P*=0.000276; Tukey's HSD, *P*=0.005681 and 0.01086) and SP samples (Tukey's HSD, *P*=0.001435 and 0.002733). A similar pattern was observed at 48 hpi (one-way ANOVA, *P*=0.00002208; Tukey's HSD, *P*=0.0001122 and 0.002874 for comparison between controls versus SS and SS/SP, respectively, and *P*=0.00008173 and 0.001674 for comparison between SP versus SS and SS/SP, respectively). Error bars represent ±s.e.m. obtained from three biological and two technical replicates. One-way ANOVA for independent or correlated samples along with Tukey's HSD test was used to confirm statistical significance in gene expression. *P≤0.05.

To determine how SP and SS impact the function of the protective epithelial barrier and potential defensive responses of the VF mucosa, we evaluated mucin 1 (Muc1) production, expression of the inflammatory cytokines IL6 and IL8 (encoded by *CXCL8*), and epithelial permeability. Specific to mucus secretion at 24 and 48 hpi, we observed significant upregulation of *MUC1* in the SP group compared to its expression in the control and SS groups (one-way ANOVA with Tukey's HSD test, *P*=0.00897 for 24 hpi and 0.004007 for 48 hpi) (Fig. 4A). *MUC1* expression was slightly reduced in SS/SP co-cultures in comparison to SP cultures for both time points; however, the difference in the expression was not statistically significant. Increased deposition of Muc1 in SP cultures at 24 and 48 hpi was also confirmed by immunofluorescent anti-Muc1 staining (Fig. 4C-E). However, in the SS group alone (Fig. 4F) and in SS/SP co-cultures at 48 hpi (Fig. 4G), the anti-Muc1 staining appeared similar to that in controls (Fig. 4B). For the mucosal immune response (Fig. 4H-J), exposure to SP significantly stimulated *IL6* expression in VF mucosal cells at 24 hpi (one-way ANOVA, *P*=1.715×10^−7^) and 48 hpi (one-way ANOVA, *P*=0.00001486) compared to that in controls (Tukey’s HSD test, *P*=3.248×10^−7^ and *P*=0.0001074 at 24 and 48 hpi, respectively), SS (Tukey’s HSD, *P*=5.218×10^−7^ and 0.00003834 at 24 and 48 hpi, respectively) and SS/SP co-cultures (Tukey's HSD, *P*=4.9117×10^−7^ and 0.00004152 at 24 and 48 hpi, respectively) (Fig. 4H). A similar pattern was observed for *IL8* expression (Fig. 4I). For the 24 hpi time point, we detected a significant increase in *IL8* expression in the SP group (one-way ANOVA, *P*=2.68×10^−12^) compared to that in controls (Tukey’s HSD, *P*=3.705×10^−13^), SS groups (Tukey's HSD, *P*=0.000003323) and SS/SP co-cultures (Tukey's HSD, *P*=3.076×10^−13^). For the 48 hpi time point, notably, we found a significant upregulation of *IL8* (one-way ANOVA, *P*=0.000139) in the SP group compared to that in controls (Tukey's HSD, *P*=0.00476) and in the SS group (Tukey's HSD, *P*=0.00009586), but not for SS/SP co-cultures (Fig. 4I). In addition to the release of cytokines, we also evaluated the expression levels of *MMP2* (Fig. 4J). Among other functions, this eukaryotic metalloprotease can assist in shedding of apical cells in stratified epithelia (Wiechmann et al., 2014). We found significant upregulation of *MMP2* in the SS and SS/SP groups compared to that in controls and SP samples for 24 hpi (one-way ANOVA, *P*=0.000276; Tukey's HSD, *P*=0.005681 and 0.01086 for comparison between controls versus SS and SS/SP, respectively, and *P*=0.001435 and 0.002733 for comparison between SP versus SS and SS/SP, respectively). A similar pattern was observed at 48 hpi (one way ANOVA, *P*=0.00002208; Tukey's HSD, *P*=0.0001122 and 0.002874 for comparison between controls versus SS and SS/SP, respectively, and *P*=0.00008173 and 0.001674 for comparison between SP versus SS and SS/SP, respectively) (Fig. 4J).

**Fig. 5.**
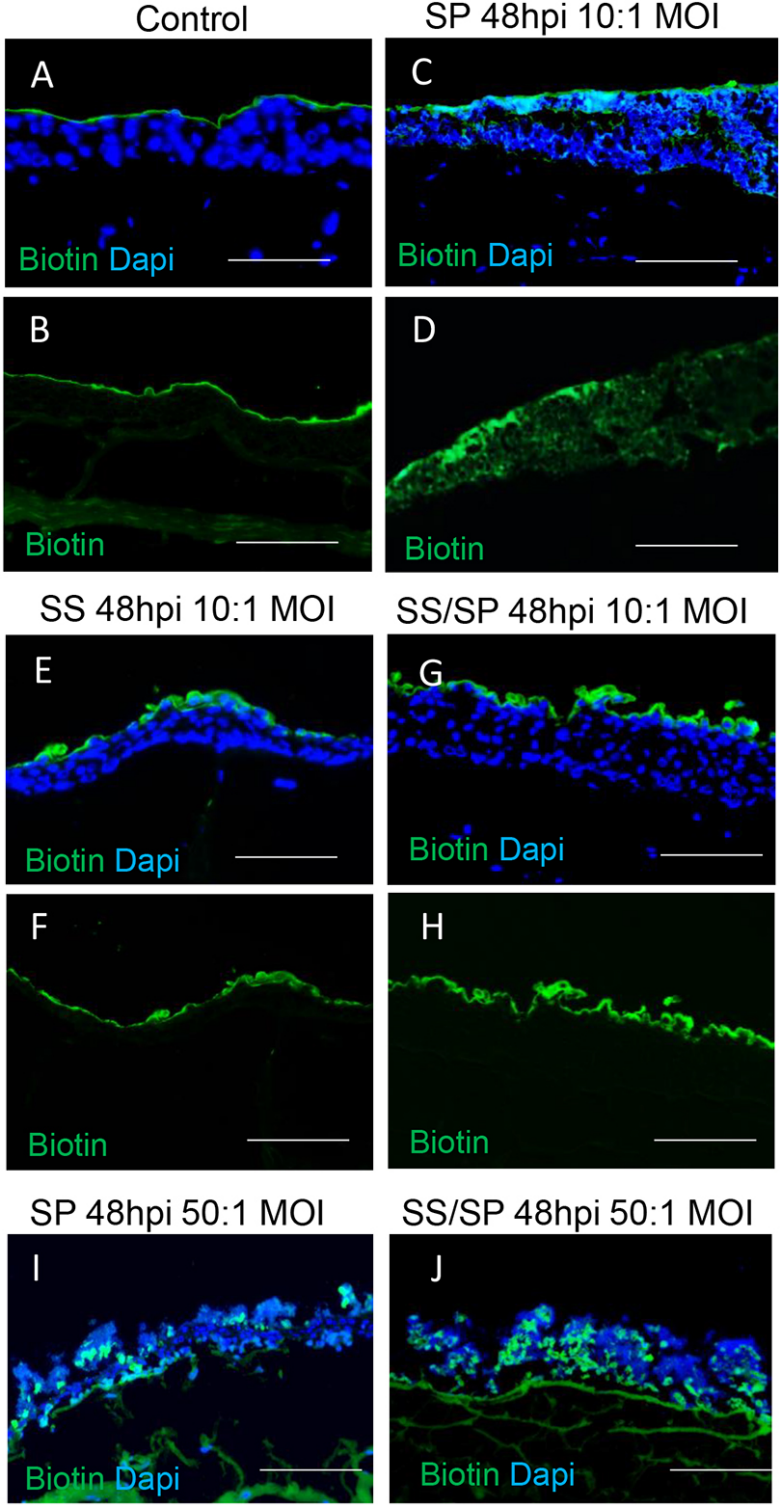
**Assessment of vocal fold epithelial barrier integrity exposed to bacterial challenges.** (A-H) Biotin epithelial permeability assay in control VF mucosae (A,B) and VF mucosae exposed to a low dose of SP only (C,D), SS only (E,F) and SS/SP co-cultures for 48 h (G,H). (I,J) Biotin permeability assay in VF mucosae exposed to a high dose of bacteria for 48 h. Scale bars: 100 µm. Images are representative of two independent experiments.

Finally, we assessed the structural integrity of the VF epithelium and conducted a biotin permeability assay. Our data show that in control samples, biotin was retained on the apical cellular surface in the form of a thin layer, demonstrating the preservation of the epithelial barrier (Fig. 5A,B). In VF mucosae exposed to a low dose of SP, biotin diffused deeper into the epithelium, indicating that the integrity of the epithelial barrier had been compromised (Fig. 5C,D). In the SS and SS/SP groups, biotin appeared to be trapped in apical cell layers, exhibiting a propensity to desquamate with an overall intact epithelium (Fig. 5E-H). Exposure of the VF epithelium to high doses of bacteria resulted in the impairment of the epithelial barrier; the epithelium became leaky, allowing biotin to pass through the epithelium into the collagen matrix with fibroblasts (Fig. 5I,J).

### The potential mechanisms SP utilizes to disrupt the structural integrity of the VF epithelium

**Fig. 6.**
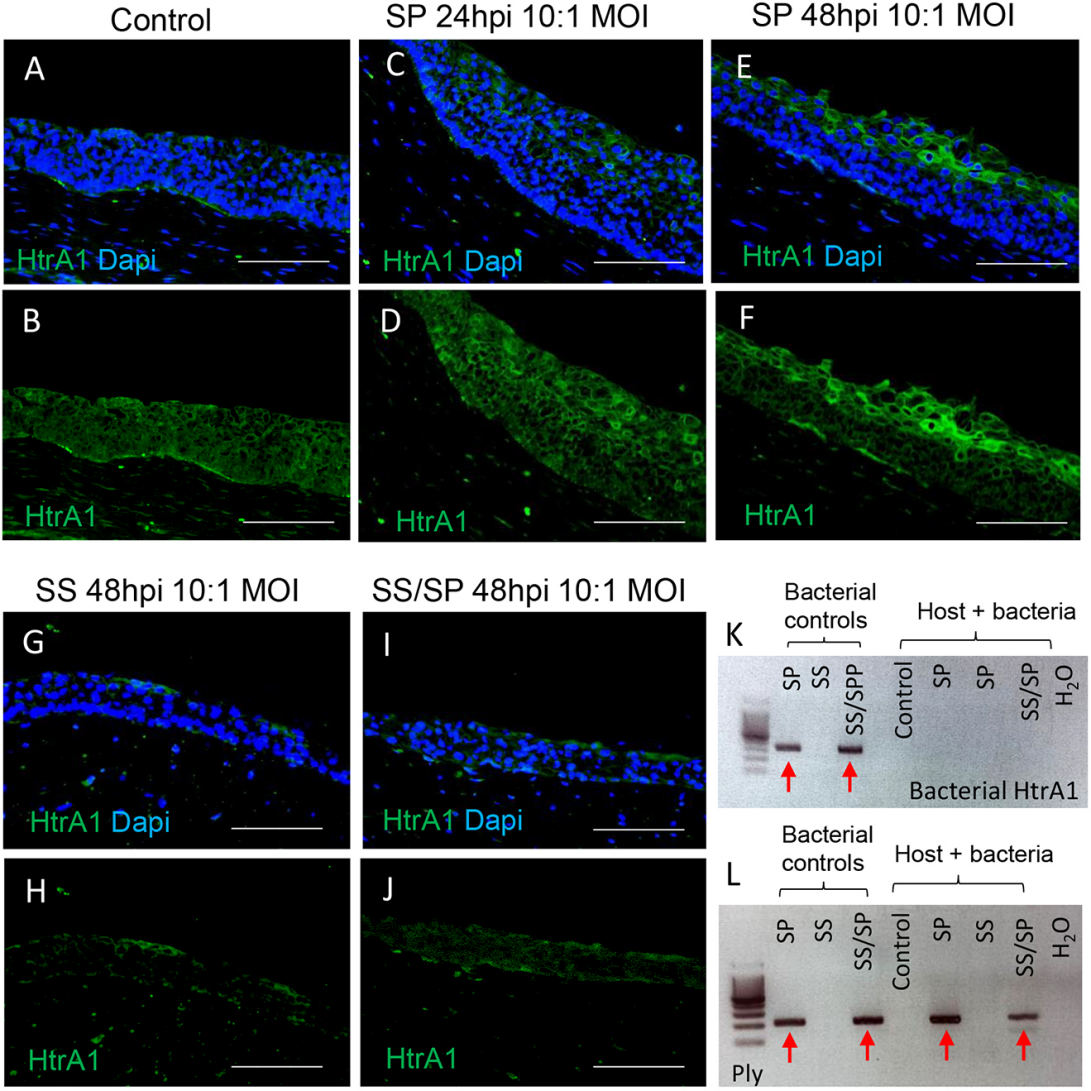
**The potential mechanisms used by SP to interact with human vocal fold epithelial cells.** (A-J) Anti-HtrA1 staining (green) in VF mucosae in control (A,B) and experimental groups exposed to a low dose of SP only (C-F), SS only (G,H) and SS/SP co-cultures (I,J) for 48 h. Scale bars: 100 µm. (K,L) Agarose gel electrophoresis visualizing bacterial HtrA1 and pneumolysin (Ply) gene PCR products in bacterial controls and VF mucosal/bacterial cell lysates. Red solid arrows denote active HtrA1 (K) and Ply (L) production. Images are representative of three independent experiments (HtrA1 staining) and two independent experiments (PCR and agarose gel electrophoresis).

To address the potential mechanisms SP utilized to disrupt the integrity of the VF epithelium, we evaluated the expression of two potential virulent exported products identified in the SP core genome, HtrA1 and Ply (Garriss et al., 2019). Immunohistochemistry (IHC) indicated a progressive rise in HtrA deposition within the SP groups from 24 to 48 hpi, compared to that in controls, SS and SS/SP groups at 48 hpi (Fig. 6A-J), with the slight staining seen in the control group as well (Fig. 6A,B). This increase in HtrA production aligns with the observed reduction of E-cad in the uppermost cell layers as shown previously (Fig. 1). We used PCR to verify that SP bacteria actively produce HtrA1, when cultured alone or together with SS (Fig. 6K). Next, we evaluated the expression of the cytotoxin Ply. We found active Ply production in \our SP and SS/SP bacterial controls, and in our SP- and SS/SP-inoculated mucosal cell lysates, but not in the controls or SS-alone groups (Fig. 6L). Interestingly, we found that Ply expression was reduced in SS/SP co-cultures compared to that in the SP-alone group or bacterial controls (Fig. 6L). Taken together, these data suggest that both HtrA1 and Ply may be involved in the infection process employed by SP, and their effects are likely neutralized in the presence of SS.

## DISCUSSION

This investigation focused on understanding the interaction between SP, a prevalent bacterial species linked to benign lesions, and SS, a commensal bacterium, with human VF epithelial cells in our recently established 3D organotypic model of human VF mucosa (Lungova et al., 2019). This experimental *in vitro* system allows for significantly greater complexity in the evaluation of bacterium–host interactions compared to previous studies that had solely relied on two-dimensional (2D) submerged cell cultures (Abfalter et al., 2016). Our model has previously demonstrated its ability to replicate key aspects of VF mucosa morphology, the collagen matrix with primary VF fibroblasts and stratified VF epithelium (Lungova et al., 2019, 2022), which enabled the assessment of host–bacterial interactions at the cellular, molecular and ultrastructural levels. Moreover, the engineered VF mucosae were grown and maintained at the A/Li, allowing for direct deposition of bacterial colonies on semi-dry apical surfaces under non-submerged conditions, which closely mimics natural infection processes (Hasan et al., 2018). This environment provides a more realistic setting for studying bacterial pathogenicity, adherence to epithelial surfaces and biofilm formation.

Our findings confirm that SP functions as an opportunistic pathogen (Dupont et al., 2020; Garriss et al., 2019). Although it is capable of producing virulent products such as HtrA1 and Ply, it does not physically adhere to the VF epithelial surface. Instead, it tends to remain in close proximity to the epithelium. This reduced ability to effectively bind to the epithelial surface may lower its virulence compared to that of *S. pneumoniae*, especially in the context of causing acute pneumococcal infections. Our results support clinical observations, where SP is more frequently associated with milder infections, benign VF lesions (Jetté et al., 2016; Hanshew et al., 2014) and chronic diseases such as chronic obstructive pulmonary disease, cystic fibrosis and chronic sinusitis (Dupont et al., 2020; Garriss et al., 2019). We have further shown that SP produces a matrix that can contribute to biofilm formation. The self-produced matrix may contain bacterial DNA, messenger RNAs (mRNAs), proteins and exopolysaccharides that promote cell–cell communication and hold bacteria together to form clumps or aggregates (Maunders and Welch, 2017). The phenomenon of autoaggregation or autoagglutination (Di Martino, 2018) in SP cultures was particularly apparent when we conducted bacterial viability assays. We speculate that SP, being non-encapsulated and lacking the high-affinity pneumococcal adhesins required for effective binding (Wen et al., 2014), may utilize this autoaggregation as a protective mechanism to survive in the larynx and interact with the laryngeal/VF mucosa. As reported elsewhere, autoaggregation serves as a bacterial defense mechanism against external stresses, such as oxidative stress, nutrient starvation, temperature fluctuations and host immune reactions (Trunk et al., 2018). Biofilm with its cytoplasmic products functions as a passive virulent factor that pathogenic bacteria such as SP may utilize to initiate infections of a human host (Schulze et al., 2021).

This study revealed two substantial structural and functional alternations in VF mucosa in response to SP that can be critical in the development of VF diseases. These changes include compromised epithelial integrity and enhanced expression of pro-inflammatory cytokines, especially IL6. Such alternations may impact not only the function of the epithelial barrier, but also the underlying lamina propria, rendering it susceptible to potential pathological remodeling and chronic inflammation. It is worth noting that similar structural changes to the VF epithelium such as loosened intercellular junctions and widened intercellular spaces have been observed in patients with VF nodules, polyps (Kotby et al., 1988; Mostafa et al., 2017), Reinke's edema (Pastuszek et al., 2003) and granulomas (Martins et al., 2009). Although phonotrauma is likely the main cause of benign lesions, our research indicates that bacterial communities could also be a key factor, particularly when a bacterial shift towards SP is evident in patients with benign lesions (Jetté et al., 2016; Hanshew et al., 2014).

We further explored how SP might impact the VF mucosa by studying two exported bacterial products, HtrA1 and Ply toxin, that have the ability to diffuse to the host epithelial surface (Boehm et al., 2015). HtrA1 is an actively secreted heat shock-induced serine protease that has a primarily protective function. HtrA is critical for stress tolerance and survival of most bacteria (Boehm et al., 2015; Ibrahim et al., 2004; Pallen and Wren, 1997). A *S. pneumoniae* strain lacking the HtrA gene exhibited reduced fitness in a competitive model of colonization (Ibrahim et al., 2004). However, as reported elsewhere, this product can also act as a virulence factor by cleaving the extracellular domain of E-cad on epithelial cells (Bernegger et al., 2022). Our histology supports prior investigations emphasizing the role of HtrA as a ‘sheddase’, an enzyme that cleaves the extracellular domain of membrane-bound proteins, resulting in the release (or ‘shedding’) of the extracellular domain into the extracellular space (Bernegger et al., 2022). Other human pathogens, such as *Helicobacter pylori*, *Campylobacter jejuni*, *Salmonella enterica*, enteropathogenic *Escherichia coli*, *Proteus mirabilis*, *Yersinia enterocolitica* and *S. pneumoniae* also exploit HtrA to cleave E-cad during their colonization (Ibrahim et al., 2004; Bernegger et al., 2022). It is important to mention that HtrA can also be produced by human cells (Chien et al., 2009). This finding may account for the faint staining observed in our control samples.

Ply is a cholesterol-dependent cytotoxin that forms lytic pores in host membranes and mediates pneumococcal disease pathogenesis (Rai et al., 2016). It can activate the migration of immune cells and production of pro-inflammatory cytokines, leading to release of reactive oxygen and nitrogen species that damage host tissues (Rai et al., 2016; Cockeran et al., 2001). Purified Ply can directly cause vascular leakage and edema (Witzenrath et al., 2006). Ply toxin is mostly found in the cytoplasm of bacterial cells and cannot be actively secreted due to the absence of secretory signals (Walker et al., 1987). It is released into the extracellular space during bacterial cell lysis, leading to the discharge of the toxin alongside the Ply gene (Salo et al., 2016). We successfully detected active Ply production in SP and SS/SP bacterial controls, as well as in our mucosal cell/SP and mucosal cell/SS/SP lysates, confirming its potential role in VF epithelial damage and enhanced production of pro-inflammatory cytokines. Notably, Ply expression was reduced in mucosal cell/SS/SP lysates, likely due to the protective function of SS. In contrast, active HtrA was only observed in our bacterial controls. Detecting HtrA via RNA isolation, cDNA conversion and PCR in our mucosal cell/bacterial lysates proved challenging. Because of its protective function, HtrA may require the presence of a sufficient number of live bacteria with actively producing HtrA protease, which is secreted into the extracellular space. Ply, however, can get into extracellular space only during autolysis. As a toxin, its DNA and RNA transcripts are likely preserved and therefore can be detected in the plasma of patients with pneumococcal infection (Salo et al., 2016). The susceptibility of SP to autolysis has been confirmed in our bacterial viability assay and correlates with the observed low abundance of SP detected by FISH, TEM and the successful amplification of Ply gene transcripts.

Next, we focused on assessment of the host defense mechanisms. Internal epithelia, including the VF stratified squamous epithelium, employ several defensive mechanisms that hinder pathogen entry and colonization. Epithelial cells secrete a viscous fluid called mucus, which contains many glycoproteins called mucins (Levendoski et al., 2014). Microorganisms coated in mucus may be prevented from adhering to epithelium. Consistent with these findings, we observed a notable increase in Muc1 expression in VF mucosal cells when inoculated with SP in comparison to its expression in controls, as determined by both qPCR analysis and immunofluorescent anti-Muc1 staining. An additional host defense mechanism includes the production of chemokines and cytokines, such as IL6, IL8, GM-CSF and G-CSF, that recruit and activate phagocytic cells to eradicate bacteria or infected cells (Parker and Prince, 2011). We observed significant upregulation of *IL6* and *IL8* in VF mucosae inoculated with SP at 24 and 48 hpi, indicating an activation of VF mucosal immune reactions when facing bacterial challenges.

**Fig. 7.**
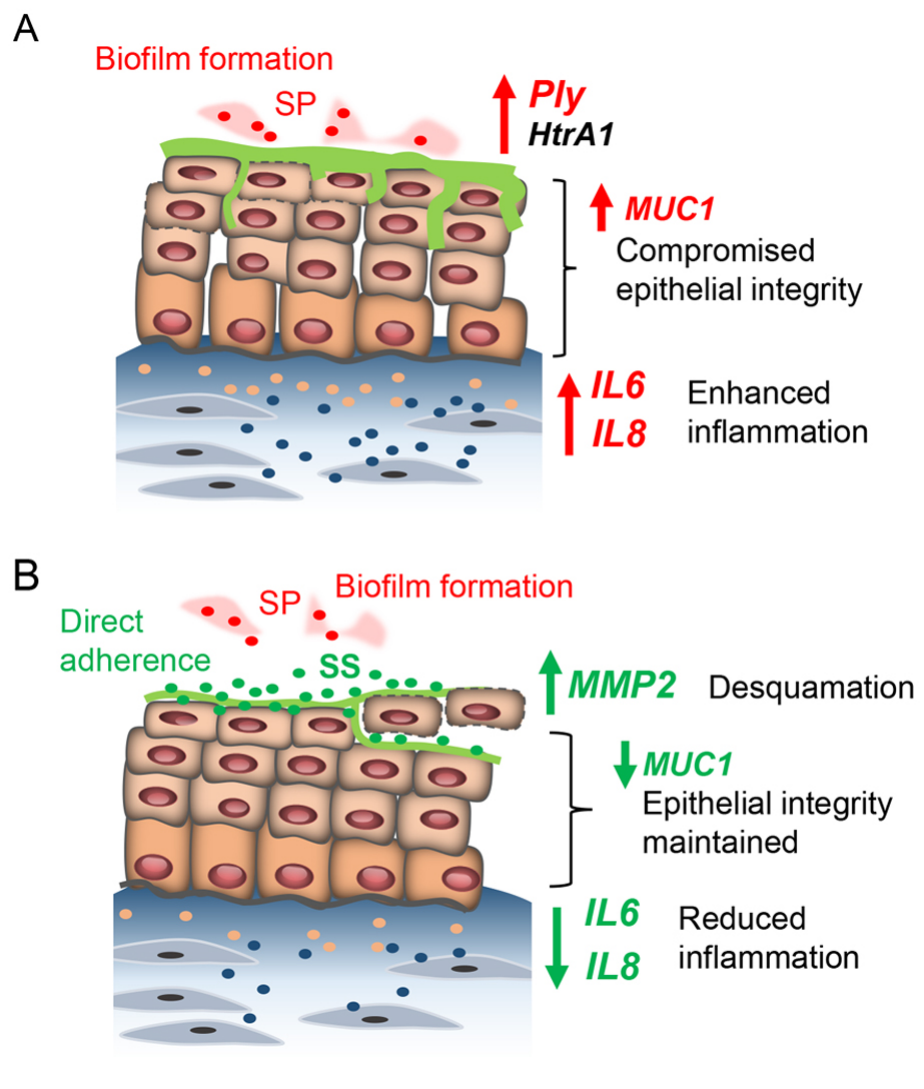
**Schematic illustration demonstrating the effects of pathogenic and commensal bacteria on the human VF mucosa.** Effects of *S. pseudopneumoniae* (SP) alone (A) and *S. salivarius/S. pseudopneumoniae* (SS/SP) co-cultures (B).

Finally, we explored the potential impact of the commensal bacterium SS on maintenance of the epithelial barrier. SS becomes a stable colonizer of oral microbiota a few days after birth and emerges as the predominant species in pharyngeal mucosa and dorsal tongue in adults (Guglielmetti et al., 2010). The ability of SS to attach to host epithelial surfaces serves as a key element contributing to its probiotic activity (Di Martino, 2018; Vertillo Aluisio et al., 2022). Our findings confirmed that, when inoculated in adequate amounts, SS adheres to the VF epithelial surface without compromising the structural integrity of the VF epithelium or triggering massive cytokine production at 24 and 48 hpi, whether inoculated alone or in combination with SP. SS can also stimulate IL8 production when cultured alone to prime VF immune responses. In our TEM analysis, we further demonstrated that, when cultured with SP, SS robustly attaches to the epithelial surface, displacing SP from the epithelial surface. This displacement can have a substantial effect on the activity of SP (Di Martino, 2018), particularly in relation to the local concentration of HtrA and Ply, leading to their diminished affinity for host receptors. Another possible probiotic benefit of SS involves triggering non-specific host defense mechanisms, such as the Mmp2-mediated desquamation of apical cells. This protective mechanism may play a vital role in effectively removing pathogens and damaged cells, thereby promoting an overall healthy VF epithelium. Apical cell desquamation may also serve as a mechanism to regulate the number of commensal bacteria that can be tolerated by the host, maintaining a balanced relationship. A schematic illustration of the effect of SP and SS/SP co-cultures on VF mucosa remodeling is provided in Fig. 7A,B. However, our research has also revealed that preserving the integrity of the VF epithelial barrier is dependent on the appropriate bacterial dosage. At high dosage, especially when SS is co-cultured with SP, commensal bacteria may react as pathogens, leading to significant epithelial damage and inflammation.

### Study limitation and future directions

Considering our findings, it is essential to address certain limitations that may impact the interpretation and generalization of our data. First, the use of a sophisticated 3D model composed of human induced pluripotent stem cell (hiPSC)-derived VF mucosa, although advantageous, comes with the limitation of hiPSC-derived cells being less differentiated than primary VF epithelial cells (Lungova et al., 2019). These hiPSC-derived cells may lack receptors crucial for successful binding of SP to the epithelial surface. To bolster extrapolation of our findings, we referred to published scientific data and clinical observations, confirming the relevance of this model in capturing essential aspects of bacterium–host cell interactions. Moreover, although hiPSC-derived, this experimental system is currently the only 3D model related to the human VF mucosa that can fully recapitulate stratification *in vitro*. Next, our current model system specifically investigated tissue homeostasis and inflammation in non-immune mucosal cells. However, we acknowledge that incorporating immune cells into this system in future experiments will enhance the accuracy of infection modeling and inflammation studies. Future investigations should also prioritize exploring the role dynamics in the VF mucosal remodeling in response to bacterial challenges, given that the larynx with VFs is a highly biomechanically active organ.

### Conclusion

In summary, this study has provided valuable insights into the effects of both pathogenic and commensal bacteria on VF mucosa structure and function, with the emphasis on the disruption or preservation of the VF epithelial barrier and modulation of VF mucosal inflammatory responses. Through a complex evaluation encompassing cellular, molecular and ultrastructural studies, this work significantly contributes to the advancement of our understanding of mucosal health and bacterial infection dynamics in the context of VF mucosal biology.

## MATERIALS AND METHODS

### hiPSC differentiation and 3D organotypic models of human VF mucosa

All stem cell work in this investigation was approved by the Stem Cell Research Oversight Committee at the University of Wisconsin-Madison (SC-2015-0008). For differentiation of hiPSC-derived VF epithelia, we used the IMR-90-4 cell line transfected with GFP (clone B10), which was validated and screened for mycoplasma contamination. To induce differentiation of hiPSCs into VF epithelia, we followed our recently published protocol (Lungova et al., 2019, 2022). hiPSCs underwent differentiation progressing through the definitive endoderm, anterior foregut endoderm and VF basal progenitors. At day 10, VF basal progenitors were reseeded on transwells of six- or 12-well plates with collagen-fibroblast constructs to establish 3D organotypic models of human VF mucosae. After reseeding, VF mucosae were cultured fully submerged in culture medium for 4 days. During this period, the culture medium was applied to transwells containing constructs and basolateral chambers of tissue culture plates. Subsequently, a transition to an A/Li was made for additional 2-3 weeks. During this phase, the culture medium was aspirated from the transwells and was applied exclusively to the basolateral chambers. We used the flavonoid adenine di-nucleotide (FAD) medium, which consisted of Dulbecco's modified Eagle medium (DMEM) and F12 in a 1:3 ratio (Gibco Life Technologies), supplemented with 2.5% fetal bovine serum (FBS; Gibco, Thermo Fisher Scientific, A5256801), 0.4 µg/ml hydrocortisone (Millipore Sigma) 8.4 ng/ml cholera toxin (Millipore Sigma), 5 µg/ml insulin (Millipore Sigma), 24 µg/ml adenine (Millipore Sigma), 10 ng/ml epidermal growth factor (R&D Systems, 236-EG) and 1% penicillin-streptomycin (Invitrogen, Carlsbad, CA, USA). Two days before inoculation, a modified FAD medium without penicillin-streptomycin was prepared. We thoroughly rinsed constructs on transwells with modified FAD medium, aspirated the remaining medium from transwells and changed the medium in the basolateral chamber only to maintain the A/Li. Human VF mucosae were inoculated with bacterial suspensions between days 32 and 39 of differentiation.

### Bacterial inoculation experiments

Human VF mucosae were inoculated with bacterial suspensions of SP only, SS only, SS/SP co-cultures and plain bacterial culture medium for controls. We used two bacterial doses: a low dose of 10:1 MOI and a high dose of 50:1 MOI (Fig. S1A). For inoculation, we employed bacterial species during the mid-logarithmic growth phase, characterized by OD values ranging from 0.4 to 0.6 nm, which corresponded to a bacterial population of approximately (2.00-3.00)×10^8^ cells per 1 ml (Fig. S1B,C). We pipetted the appropriate quantity of bacterial cells from the bacterial cell cultures into a microcentrifuge tube, spun them down, aspirated the supernatant and then resuspended the cells in 100 µl (for a six-well construct) and 20 µl (for a 12-well construct) of bacterial culture medium. For SS/SP co-cultures, we mixed a half dosage of SP with a half dosage of SS to maintain 10:1 and 50:1 MOI. Bacterial suspensions were then directly applied on the top of the constructs to maintain the A/Li. For control samples, we used plain bacterial culture medium. Modified FAD medium was changed in the basolateral chambers and VF mucosae were incubated at 5% CO_2_ and 37°C for 24 or 48 h. 24 and 48 hpi, VF mucosae were harvested and characterized by IHC, TEM, regular PCR and qPCR. Modified FAD culture medium collected from the basolateral chambers of control and experimental groups at 24 hpi was used for LDH cytotoxicity assay.

### Bacterial expansions for inoculation

Both SS and SP were cultivated and expanded in brain heart infusion (BHI) broth (Millipore Sigma). We mixed 38.75 g BHI broth powder with 1 l of water, divided the resulting solution into two bottles and autoclaved the broth, which was allowed to cool and stored at room temperature (RT) until use. For inoculation, 14 ml bacterial tubes were filled up with 6 ml of the broth. We used a 10 ml pipette to remove a small portion of bacterial colonies from glycerol stocks and placed the tips into the broth. Bacterial tubes were kept closed but not tight to allow air to get into the tube. Bacterial tubes were placed in a 37°C degree incubator and allowed to shake at 225 rpm overnight. The following day, we measured OD values using the SmartSpec Plus spectrophotometer (Bio-Rad), generated growth curves for both bacterial species following the bacterial growth curve protocols (https://microbenotes.com/bacterial-growth-curve-protocol) and expanded bacterial cultures for inoculation. Bacterial species used in this study were obtained from American Type Culture Collection (ATCC, BAA-960) for SP strain CDC-SS-1757, and Ward's Science (Rochester, NY, USA; 470179-174) for SS.

### Histology and fluorescence IHC

At selected time points (24 and 48 hpi), VF mucosae were harvested, washed with 1× phosphate buffered saline (PBS), then fixed in fresh 4% paraformaldehyde for 15 min at RT and embedded in the histogel (Thermo Fisher Scientific). Samples were then dehydrated in a series of ethanol, treated with xylene, embedded in paraffin, and cut to 5 µm thick serial sections using a Leica HistoCore BIOCUT microtome. For histology, sections were deparaffinized, rehydrated and routinely stained with Hematoxylin and Eosin. For fluorescence IHC examination, we followed standard fluorescence IHC protocols (Lungova et al., 2019, 2022). Antigen retrieval was performed by heating sections in sodium citrate, pH 6, in an 80°C water bath for 2 h. The primary and secondary antibodies in this study have been routinely used before and validated elsewhere (Lungova et al., 2019, 2022). The primary antibodies used included: rabbit anti-E-cad diluted to 1:100 (Cell Signaling Technology, 3195S), mouse anti-Muc1 diluted to 1:200 (Abcam, ab70475) and rabbit anti-HtrA1 diluted to 1:100 (Proteintech, 55011-1-AP). They were applied overnight at 4°C. The secondary antibodies used were: Alexa Fluor 488-conjugated goat anti-rabbit IgG at 1:500 (Invitrogen, A11070) and Cy3-cojugated goat anti-mouse at 1:200 (Jackson ImmunoResearch, 115-165-003). They were applied for 1 h and 30 min at RT. Sections were mounted using Fluoromount-G with DAPI (Invitrogen, 00-4959-52). Images were acquired with a Nikon Eclipse Ti2 microscope and were adjusted for brightness using the NIS-Elements software (Nikon). In our analysis, we used three biological replicates and two technical replicates for both control and experimental groups for both time points and bacterial doses.

### Giemsa staining

Paraffin-embedded sections were deparaffinized in xylene two times for 5 min, then incubated in series of ethanol – 100% ethanol two times for 5 min; 95% ethanol two times for 5 min and 70% ethanol two times for 5 min – followed by submerging slides in deionized water for 5 min. Next, the slides were fixed in methanol for 5 min and allowed to dry for 15 min on the bench. Subsequently, we stained the slides with Giemsa Stain (Modified solution; Sigma-Aldrich) diluted to 1:20 with deionized water as recommended by the supplier for 20 min at RT. The slides were then rinsed in deionized water and allowed to dry overnight to evaluate bacterial stains. Images were acquired with a Nikon Eclipse Ti2 microscope and were adjusted for brightness using the NIS-Elements software. In our analysis, we used three biological replicates and three technical replicates for both control and experimental groups for both time points.

### 16S FISH

Paraffin-embedded sections were deparaffinized in xylene two times for 10 min, incubated in 100% ethanol for 5 min and allowed to air dry on the bench. Meanwhile, the hybridization solution (15% formamide, 20 mM Tris-HCl, 0.9 M NaCl, 1% SDS and PCR-grade water) was prepared and mixed with an EUB338-DB probe (stock concentration of 100 µM; Integrated DNA Technologies) diluted to 1:100 alongside two negative controls – one with hybridization solution and EUB338 negative probe (EUB338-neg) (stock concentration of 200 µM; Integrated DNA Technologies) diluted to 1:200, and one with hybridization solution only without the probe. The probe sequences are listed in Table S1. Mixtures were then applied on sections, and sections were placed into a dehumidifying chamber filled with water and incubated at 50°C overnight. The next day, the sections were washed in fresh FISH washing buffer (20 mM Tris-HCl, 0.9 M NaCl, 1% SDS and PCR-grade water) and incubated at 50°C for an additional 10 min. Next, we co-stained for E-cad following the regular IHC protocol (Lungova et al., 2019). Images were acquired with a Nikon Eclipse Ti2 microscope and were adjusted for brightness using NIS-Elements software. We used three biological and two technical replicates in the procedure for control and experimental groups for both time points and bacterial doses.

### TEM

For TEM, VF mucosae were fixed in 2.5% glutaraldehyde and 2% paraformaldehyde in a 0.1 M sodium cacodylate buffer (pH 7.4) overnight at 4°C and processed using routine techniques. Briefly, constructs were washed in a 0.1 M sodium cacodylate buffer and postfixed in 1% osmium tetroxide in the same buffer for 2 h at RT. Tissues were dehydrated in a graded ethanol series, rinsed twice in propylene oxide, and embedded in Epon 812 epoxy resin (Polysciences) under vacuum. Finally, the samples were flat embedded between glass slides. After resin polymerization, one of the two glass slides were removed and blank resin cylinders were glued to the sections. The constructs were thin sectioned for TEM using a Leica EM UC6 ultramicrotome and stained with Reynolds’ lead citrate stain and 8% uranyl acetate in 50% ethanol to increase contrast. Sections were viewed with a Philips CM120 electron microscope and images were captured with a MegaView III side-mounted digital camera (BioSprint12, Advanced Microscopy Techniques, Woburn, MA, USA).

### Bacterial cell viability assay

To evaluate the viability of SP and SS during culturing, we first expanded the bacterial colonies as described in the ‘Bacterial expansions for inoculation’ section. Then bacterial suspensions (SP, SS and SS/SP co-cultures) at a dose of 10:1 MOI were mixed with Matrigel (Corning Matrigel Growth Factor, Sigma-Aldrich, 356230) (SP alone, SS alone, and SS/SP co-culture) to immobilize the bacteria and plated on culture dishes in drops. These were allowed to solidify in an incubator at 37°C and 5% CO_2_ for at least 15 min. Subsequently, the drops were partially flooded with FAD medium to mimic A/Li conditions. After 24 and 48 hpi, we removed bacterial cultures from the incubator, washed them with PBS and applied a fluorogenic DNA-binding dye (BactoView Dead Stain FITC, Biotinum, Fremont, CA, USA) for live/dead cell discrimination in bacteria. We followed the protocol recommended by the supplier. Stained bacterial cultures were visualized with a Nikon Eclipse Ti2 microscope. Images were adjusted for brightness using NIS-Elements software. We used two biological and four technical replicates in the procedure for control and experimental groups for both time points.

### RNA isolation from VF mucosal cells for qPCR

To isolate RNA from VF mucosal cells, collagen constructs were harvested, washed in 1× PBS twice for 5 min, placed into microcentrifuge tubes and stored at −80°C. We used the ReliaPrep RNACell Miniprep System (Promega) with mechanical collagen gel disruption and followed the manufacturer's instructions. 1000 ng of RNA (500 ng of RNA for low-yield RNA samples) was reverse transcribed to cDNA using reverse transcription reagents (Go Script, Promega) following the manufacturer's protocol. A total volume of 0.4 µl of cDNA (0.8 µl of cDNA for low-yield RNA/cDNA samples) was used per 20 µl real-time qPCR reaction using Power Up SYBR Green Master Mix (Applied Biosystems) and run for 40 cycles in duplicates on a 7500 Fast Real-Time PCR System machine (Applied Biosystems), according to the manufacturer's instructions. Gene-specific primers are listed in Table S1. Relative gene expression, normalized to β-actin expression (ΔCT) and control VF mucosae (ΔΔCT), was calculated as fold change using the 2^−ΔΔCT^ method. If undetected, cycle number 40 was assigned to allow fold-change calculations. Data are presented as the average of three biological and two technical replicates ±standard error of the mean. One-way ANOVA for independent or correlated samples analysis along with Tukey's HSD test were used to confirm statistical significance in gene expression (*P≤0.05).

### RNA isolation from bacterial controls and VF mucosal cells for PCR and agarose gel electrophoresis

To isolate RNA from bacterial controls, we used bacterial cultures cultivated in Matrigel drops and partially flooded with FAD medium as described in the ‘Bacterial cell viability assay’ section. To ensure an adequate RNA yield, we pooled at least six Matrigel drops for each condition and time point. Cells were harvested in PBS. To break down the Matrigel drops and release the cells, we vigorously pipetted the mixture and collected bacterial cells into 1.5 ml microcentrifuge tubes. Cell suspensions were spun down, the supernatant was aspirated and the bacterial cell pellet was stored at −80°C. As for VF mucosal cells, collagen constructs were harvested without washing, immediately placed into the microcentrifuge tubes and stored at −80°C. For RNA isolation, we used the Monarch Total RNA Miniprep Kit (New England BioLabs) and followed the manufacturer's instructions for tough-to-lyse samples using mechanical lysis. Eluted RNA was stored at −20°C until use. Subsequently, 500 ng of RNA (or 250 ng of RNA for low-yield samples) underwent reverse transcription to cDNA using reverse-transcription reagents (Go Script, Promega) following the manufacturer's protocol. The generated cDNA was used for PCR gene amplification targeting bacterial HtrA1 and Ply genes. Gene-specific primers are listed in Table S1. To amplify both genes, the GoTag Green Master Mix (M712) (Promega) was used per the manufacturer's instructions. 1 μl of cDNA (for 500 ng RNA samples) and 2 μl of cDNA (for 250 ng RNA samples) were used per reaction with the following amplification conditions: 94°C for 15 min; 40 cycles of 94°C for 1 min, 50°C for 1 min, 72°C for 1 min; followed by 72°C for 5 min. PCR products were electrophoresed on a 1.5% agarose gel containing ethidium bromide.

### Biotin permeability assay and cryosections

A tracer permeability assay using the surface biotinylation technique was performed according to the method developed by Chen et al. (1997). This method makes use of the biotinylation that stably crosslinks proteins and does not pass through the intact cell junction (Suzuki et al., 2018). We first harvested VF mucosae at 48 hpi and washed them briefly with 1× PBS. The mucus covering the surface of the VF mucosa was removed by washing constructs five times with Hank's balanced salt solution (HBSS) containing 1 mM CaCl_2_. Then 1 ml of 1 mg/ml EZ-Link SulfoNHS-LC-Biotin (Thermo Fisher Scientific) in HBSS containing 1 mM CaCl_2_ was applied on top of constructs for 10 min (100 µl per a six-well plate construct and 20 µl per a 12-well plate construct), followed by one wash with HBSS containing 1 mM CaCl_2_. Constructs were then embedded in optical cutting temperature compound (OCT) (Tissue-Tek, Kyoto, Japan) and samples were stored at −80°C until use. Before cryosectioning, blocks were removed from the freezer, allowed to warm up to −21°C in a cryostat (Leica CM3050S) and cut to 8 µm-thick sections (chamber temperature, −21°C, and objective temperature, −21°C). Sections were collected on pre-coated slides and stored at −80°C until use. The distribution of biotin was visualized by incubating the cryosections with anti-biotin-FITC (Miltenyi Biotec, Bergisch Gladbach, Germany; clone Bio3-18E7; 130-113-852) following our IHC protocol. Anti-biotin FITC was diluted to 1:100 and applied overnight at 4°C. The next day, sections were washed in 1× PBS three times for 5 min and mounted with Fluoromount-G with DAPI. Images were acquired with a Nikon Eclipse Ti2 microscope and were adjusted for brightness using the NIS-Elements software. We used two biological and two technical replicates in the procedure for control and experimental groups for both bacterial doses, at 48 hpi only.

### LDH assay for cytotoxicity

To evaluate the cytotoxic effect of SP and SS on VF mucosal cells, LDH release was measured. After 24 h of incubation with bacterial species, modified FAD medium from the basolateral chambers was collected. Levels of LDH were assayed in duplicates using an LDH Glo Cytotoxicity Detection Kit (Promega) according to the manufacturer's instructions. We first created a LDH positive control standard curve using reagents provided by the supplier to show that relative luminescence values from our control and experimental groups fall within the linear range of the LDH positive control standard curve (Fig. S1D). To calculate the percentage of cytotoxicity in our control and experimental samples, we included the positive control with the total 100% LDH release and the background level (0% LDH release) represented by the modified FAD culture medium. For the positive control, modified FAD medium from VF mucosal cells exposed to 1% Triton X-100 for 15 min was used in the experiment. Luminescence was recorded 60 min after adding LDH detection reagents using a FlexStation 3 microplate reader (Molecular Devices) and the percentage of toxicity was calculated as:

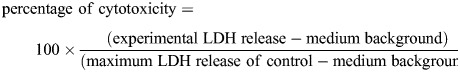
We used thee biological and two technical replicates in the analysis for both bacterial doses. One-way ANOVA for independent or correlated samples along with Tukey’s HSD test were used to confirm statistical significance in the percentage of toxicity (*P≤0.05).

### Writing the manuscript

During writing, we used Grammarly to correct grammar mistakes and spelling issues.

## Supplementary Material

10.1242/dmm.050670_sup1 Supplementary information
