# Supplementary material for: Contribution of Streptococcus pseudopneumoniae and Streptococcus salivarius to vocal fold mucosal integrity and function
Source: Dis Model Mech. 2024 Jul 16;17(7):dmm050670. doi: 10.1242/dmm.050670 (PMC11273296; doi:10.1242/dmm.050670)
Supplement: Supplementary information [file dmm-17-050670-s1.pdf]

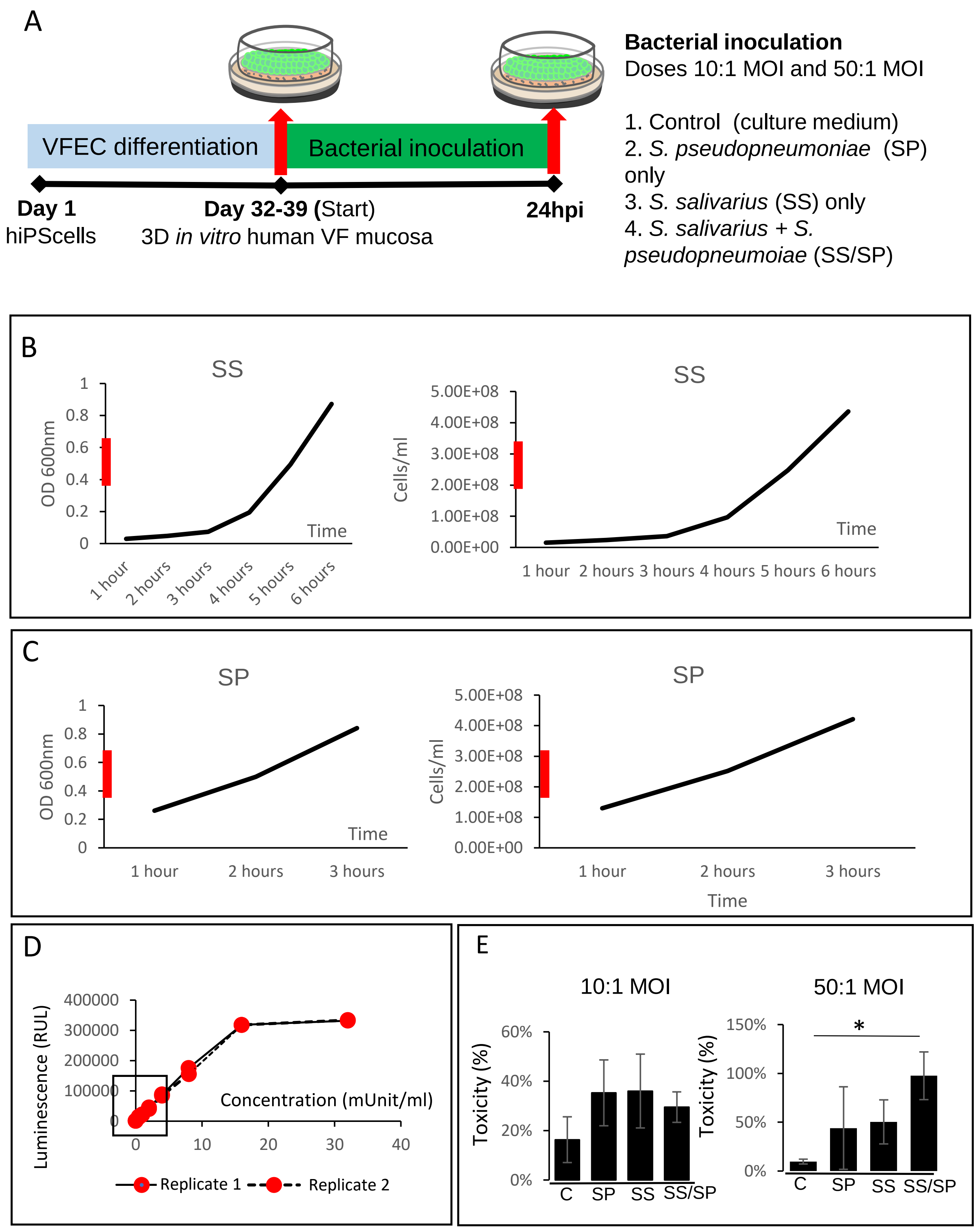

Disease Models & Mechanisms • Supplementary information

Fig. S1. Cytotoxicity assay and bacterial species used in the study for inoculation. Schematic illustration of experimental design for Cytotoxicity Assay (A). HiPS cells were first differentiated into VF basal progenitors that were re-seeded on collagen-fibroblast constructs to create a three-dimensional model of human VF mucosa. The developed model was then inoculated with bacterial species between 32 and 39 days for 24h. We included control VF mucosae inoculated with plain bacterial growth medium (1) and three experimental groups exposed to *S. pseudopneumoniae* only (2), *S. salivarius* only (3) and co-cultures of *S. pseudopneumoniae* and *S. salivarius* (4), in the experiment. We used two bacterial doses: a low dose of 10:1 MOI and a high dose of 50:1 MOI. The *S. salivarius* growth curve as represented by the OD (optical density) values and bacterial cell numbers per 1ml (B). The *S. pseudopneumoniae* growth curve as represented by the OD (optical density) values and bacterial cell numbers per 1ml (C). Linear range of LDH positive control standard curve. RLU, relative luminescence units

(D). Percent toxicity calculated for both bacterial doses 24h post-inoculation (E). Error bars represent ± standard error of the mean obtained from three biological and two technical replicates. One-Way ANOVA of variance for independent or correlated samples statistical analysis along with Tukey HSD test were used to confirm statistical significance in Percent Cytotoxicity, *p-value* ≤ 0.05 (*).

Disease Models & Mechanisms • Supplementary information

Table S1. Gene primer and EUB338 probe sequences used in this study.

Gene<br>Probe	Forward (5'-3')	Reverse (5'-3')
**Beta-Actin**	ACGTTGCTATCCAGGCTGTGCTAT	CTCGGTGAGGATCTTCATGAGGTAGT
**Cdh1**	CGATTAAAGGTGGAGAGAGGACTG	AATGAATGGTGGACAGACACAGG
**Muc1**	AGACGTCAGCGTGAGTGATG	GACAGCCAAGGCAATGAGAT
**IL6**	AAGCCAGAGCTGTGCAGATGAGTA	GCTGCGCAGAATGAGATGAGTTGT
**IL8**	AGACATACTCCAAACCTTTCCACCC	TCCAGACAGAGCTCTCTTCCATCA
**Mmp2**	AGAAGGATGGCAAGTACGGCT	AGTGGTGCAGCTGTCATAGGATGT
**Ply**	CCCACTCTTCTTGCGGTTGA	TGAGCCGTTATTTTTTCATACTG
**HtrA1**	ATGAAACATCTAAAAACATT	CGCCAGATCCCTCACTAGAG
**EUB338** -DB	/5Cy3/GCTGCCTCCCGTAGGAGT/3Cy3Sp/	
**EUB338** - neg	/5Cy3/ACTCCTACGGGAGGCAGC	

Disease Models & Mechanisms • Supplementary information
